# Controlled induction of DNA double-strand breaks in the mouse liver induces features of tissue ageing

**DOI:** 10.1038/ncomms7790

**Published:** 2015-04-10

**Authors:** Ryan R. White, Brandon Milholland, Alain de Bruin, Samuel Curran, Remi-Martin Laberge, Harry van Steeg, Judith Campisi, Alexander Y. Maslov, Jan Vijg

**Affiliations:** 1 Department of Genetics, Albert Einstein College of Medicine, 1301 Morris Park Avenue, Bronx, New York 10461, USA; 2 Faculty of Veterinary Medicine, Department of Pathobiology, Dutch Molecular Pathology Center, Utrecht University, Yalelaan1, 3584 CL Utrecht, The Netherlands; 3 Buck Institute for Research on Aging, 8001 Redwood Boulevard, Novato, California 94945, USA; 4 National Institute of Public Health and the Environment (RIVM), Antonie van Leeuwenhoeklaan 9, MA 3721 Bilthoven, The Netherlands; 5 Lawrence Berkeley National Laboratory, 1 Cyclotron Road, Berkeley, California 94720, USA

## Abstract

DNA damage has been implicated in ageing, but direct evidence for a causal relationship is lacking, owing to the difficulty of inducing defined DNA lesions in cells and tissues without simultaneously damaging other biomolecules and cellular structures. Here we directly test whether highly toxic DNA double-strand breaks (DSBs) alone can drive an ageing phenotype using an adenovirus-based system based on tetracycline-controlled expression of the SacI restriction enzyme. We deliver the adenovirus to mice and compare molecular and cellular end points in the liver with normally aged animals. Treated, 3-month-old mice display many, but not all signs of normal liver ageing as early as 1 month after treatment, including ageing pathologies, markers of senescence, fused mitochondria and alterations in gene expression profiles. These results, showing that DSBs alone can cause distinct ageing phenotypes in mouse liver, provide new insights in the role of DNA damage as a driver of tissue ageing.

## Abstract (web summary)

Accumulation of DNA damage is a hallmark of cellular ageing but cause and effect are unclear. Here White *et al.* induce clean DNA double-strand breaks in the liver of mice using a modified restriction enzyme and demonstrate that DNA damage alone is sufficient to recapitulate some aspects of tissue ageing.

## Graphical abstract

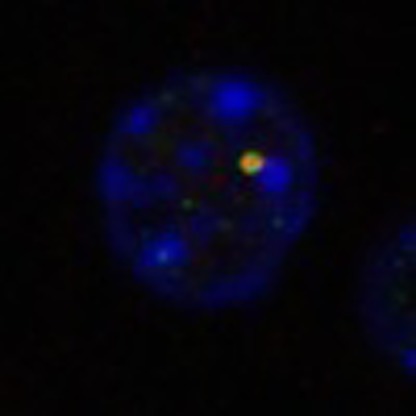

DNA double-strand breaks (DSBs) are one of many types of DNA damage that occur spontaneously in all living organisms. DSBs can be induced by ionizing radiation, radiomimetic chemicals or reactive oxygen species, but also during DNA replication when a polymerase encounters a single-strand lesion at a replication fork^1^. DSBs pose problems for cells because their immediate and efficient repair by ligation is often constrained by their physical separation and/or the need to process damaged DNA termini^2^,^3^. DSBs are repaired primarily by either homologous recombination or non-homologous end-joining. Homologous recombination is an error-free pathway that utilizes sites of sequence homology, usually a sister chromatid, to repair breaks^4^. Non-homologous end-joining is error prone, has no requirement for homology and frequently causes deletions, insertions and translocations^5^. In the absence of repair, damaged cells can be eliminated by apoptosis. Alternatively, mitotically active cells can respond to DSBs by becoming senescent, the permanent cessation of cell division. DSBs can result in genome rearrangements, when multiple DSBs in the same cells are annealed erroneously^6^. Thus, DSBs are highly toxic lesions that can promote cancer and, possibly, ageing^7^.

DSBs have been implicated in ageing, through cell loss, the accumulation of senescent cells^8^ or genome rearrangements^9^. Interestingly, mammals show an age-related increase in foci of phosphorylated H2AX, a marker of DSBs, in various organs and tissues^10^,^11^. Such foci may stem from the decreased propensity of a DSB to be repaired as a function of age^12^, or may reflect an accumulation of senescent cells, which harbour persistent DNA damage foci^13^. In addition, DSBs have been indirectly linked to ageing through the use of DSB repair-deficient mouse models, such as *ERCC1-* and *Ku80-*deficient mice. Mice harbouring such DSB repair defects display multiple symptoms of premature ageing and have a reduced lifespan^14^,^15^. In humans, signs of premature ageing have been observed in adult survivors of childhood cancer who had been treated with agents known to induce DSBs, such as ionizing radiation and a variety of chemotherapeutic drugs^16^,^17^. However, it remains unclear whether these premature ageing phenotypes are truly an effect of DSBs or collateral damage to other molecules such as lipids and proteins. To establish a definitive causal relationship between DSBs and ageing, experimental animal models are needed in which DSBs can be specifically induced in cells and tissues.

Here we use an adenoviral construct encoding the SacI restriction endonuclease inducible by doxycycline (DOX), previously described by us^18^, to directly test, for the first time, the possibility that DSBs alone can cause phenotypes associated with ageing. The results indicate that one to two months after inducing DSBs in the liver, young mice show multiple symptoms of ageing similar to those seen in untreated livers of normally aged control mice.

## Results

### Induction of DSBs by SacI adenovirus

**Figure 1:**
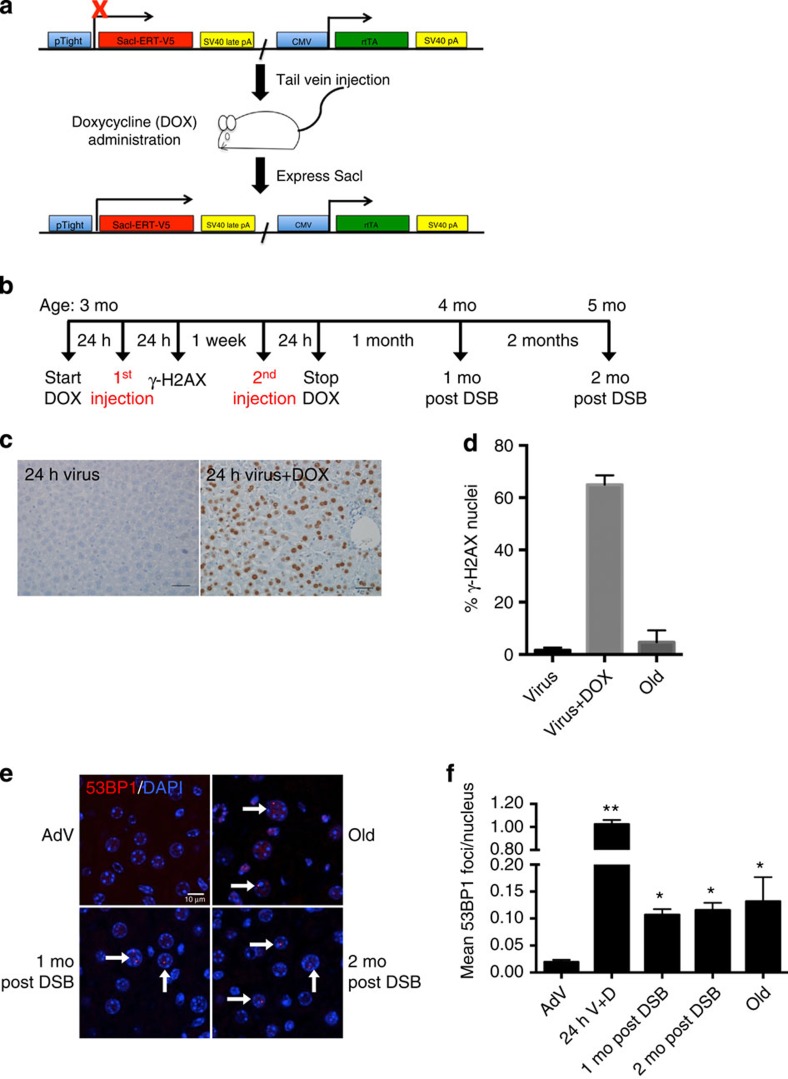
DSB induction by SacI adenovirus. (**a**) Schematic of the SacI adenoviral construct (SacI AdV) and its activation by DOX. (**b**) Schematic of the experimental timeline and mouse ages at which DSBs were induced. (**c**) Liver sections were immunostained for γ-H2AX 24 h after SacI AdV tail-vein injection either without (left panel) or with (right panel) DOX administration, and old (28 months) mice as a control. Scale bar, 100 μM. (**d**) Results were quantified and data shown as mean percentage of nuclei positively stained±s.d.; Virus *n*=3, Virus+Dox *n*=2, Old *n*=3. (**e**) Representative images of liver sections stained with 53BP1 (red) and DAPI (blue), analysed by confocal microscopy. White arrows indicate persistent 53BP1 foci. Scale bar, 10 μM. (**f**) Mean number of 53BP1 foci per nucleus was quantified. Data shown are the mean±s.e.m. of foci per nucleus, where AdV, 1 mo, 2 mo post DSB *n*=4, 24 h V+D *n*=2 and old *n*=3; >300 nuclei were scored per animal. *P* values were calculated using Student’s unpaired *t*-test to AdV samples. *P<0.05, **P<0.01. Mo, months.

We previously described a system for the quantitative introduction of DSBs in mammalian cells^18^. The system consists of an adenoviral vector (AdV) containing a tetracycline-inducible, composite SacI restriction endonuclease, which is fused to a mutant oestrogen receptor and a constitutively expressed reverse transactivator (rtTA) gene (Sac1 AdV; Fig. 1a). SacI recognizes a 6-bp palindromic sequence that occurs in the mammalian genome an estimated 1.3 million times, with ∼130,000 sites expected to be available for cleavage in the context of chromatin^18^. SacI binding and cleavage generates cohesive sticky ends, which should be easily religated. However, a few of these ends can be eroded by endogenous exonuclease activities, thereby preventing religation, creating a DSB and activating the DNA damage response. In cultured cells, DNA restriction endonucleases have been shown to be mutagenic^19^.

We injected the SacI AdV into the tail veins of young, 3-month-old mice, 24 h after administering DOX in their drinking water (Fig. 1b). The AdV treatment was repeated 1 week later, after which the mice were allowed to recover for a 1- and 2-month period before killing. In parallel, young control animals received the SacI AdV without DOX (AdV control). As a positive control for ageing phenotypes, we used naturally aged, 28-month-old mice, which were killed at the same time. We specifically chose to analyse changes in the liver, as intravenous administration of an adenovirus vector results mostly in hepatocyte transduction^20^.

To confirm the induction of DSBs mostly in the liver, we immunostained for γ-H2AX 24 h after the first SacI AdV injection. Approximately 65% of hepatocytes in DOX-treated mice, compared with only 2% of hepatocytes in AdV control mice, contained nuclei that stained positively for γ-H2AX (Fig. 1c,d). Analysis of naturally aged mice showed a significant elevation of the frequency of γ-H2AX-positive cells up to ∼9%, corroborating data from others^10^. For comparison, we also stained five other tissues for γ-H2AX after adenoviral injection and DOX treatment (Supplementary Fig. 1), showing that liver is by far the most robustly targeted tissue using our AdV, followed by a small portion of γ-H2AX-positive cells in pancreas.

We also quantified DSBs in liver by staining for 53BP1 (Fig. 1e), which confirmed the γ-H2AX results at 24 h after adenoviral injection and DOX, but also allowed counting the average number of DSBs as foci per nucleus. In our hands, 53BP1 foci have a much better resolution for that purpose than γ-H2AX foci. The results indicated approximately one DSB on average per hepatocyte 24 h after injection compared with ∼0.02 foci in the untreated control mice (Fig. 1f). Most of these 53BP1 foci disappeared, presumably due to rapid repair and/or apoptosis, but some remained. Indeed, at 1 and 2 months after the AdV infections, the levels were still significantly elevated in comparison with AdV control mice, that is, 0.10 and 0.11 foci at 1 and 2 months, respectively, as compared with 0.02 in the controls. The levels at 1 and 2 months after treatment were about the same as what was observed in naturally aged, 28-month-old mice. These results confirm that after an initially high level, DSB numbers in the liver of treated mice quickly decrease to physiological levels, comparable to what was observed in aged mice.

### DSBs induce multiple normal liver ageing pathologies

Organ-specific patterns of multiple pathology are hallmarks of the ageing process in many animal species. In the mouse, we and others have observed a set of pathological lesions that greatly increase in frequency with normal ageing^15^,^21^,^22^. To determine whether DSBs induce these same pathologies at early age, we performed haematoxylin and eosin staining on all liver sections from young treated with DOX and SacI adenovirus, SacI adenovirus alone, young and old control mice, after which two independent pathologists, from different pathology centres, performed histopathological analysis in a blinded manner. Typical ageing pathologies were scored based on a scale of 0–5, with 0 being absent and 5 being most severe. The mean value±s.e.m. was calculated for all samples.

**Figure 2:**
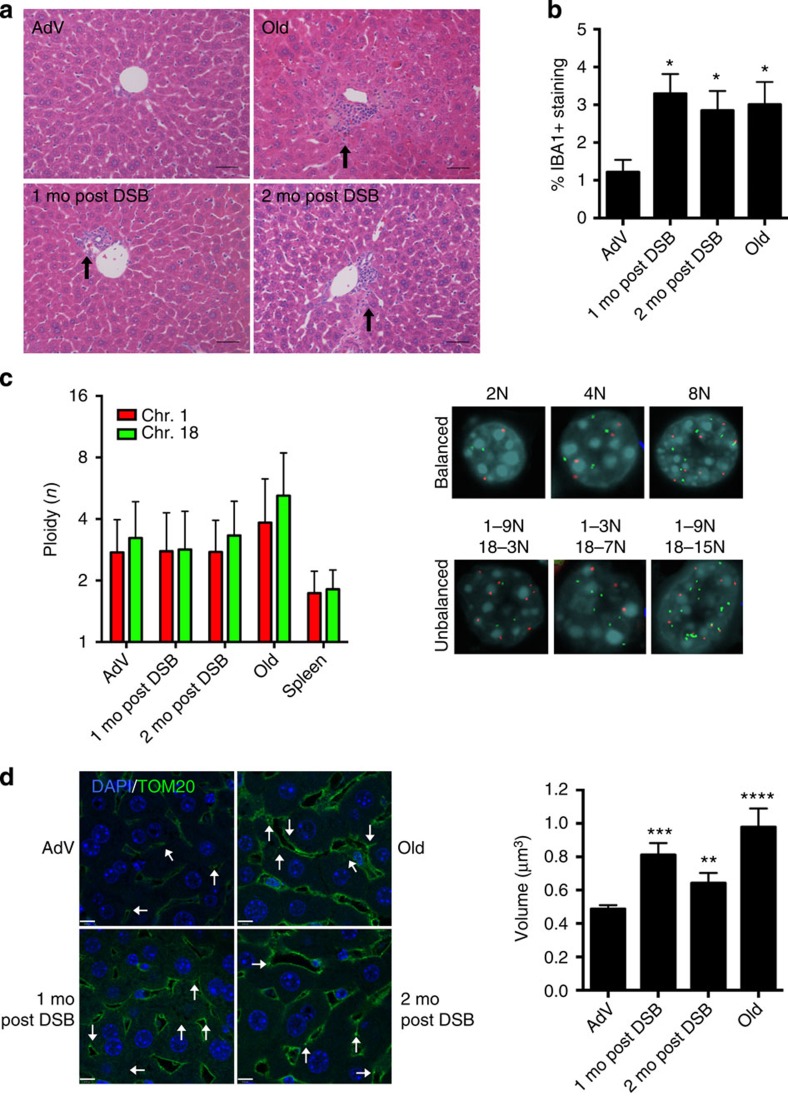
Phenotypic analysis of DSB-induced mouse liver. (**a**) Representative haemotoxylin and eosin-stained liver sections (portal vein orientation) assessed blinded for pathological characteristics of aging at × 20 magnification. Black arrows indicate sites of lymphocytic infiltrates. (**b**) Quantification of activated macrophages determined by percentage of IBA1 staining. Data shown represents the mean±s.e.m. from three images per *n*, where *n*=4 for all cohorts. (**c**) Dual-colour interphase FISH was performed on liver sections. Ploidy for chromosome 1 (red) and chromosome 18 (green) was determined for 100 hepatocytes per *n* where *n*=3 for all cohorts. The ploidy of each chromosome was plotted as mean±s.d. Representative images are shown for cells with balanced chromosome ploidy for 2, 4 and 8 N and unbalanced cells. (**d**) Mitochondrial volume was quantified by immunofluorescent staining for TOM20 (green), a mitochondrial membrane-bound protein and nuclei (blue) and analysed by confocal microscopy. White arrows indicate analysable mitochondria after background noise subtraction from *z*-stack. Mean volume (in *x*–*y*–*z* planes) was calculated for eight images per *n* (>2,000 mitochondria), where AdV, 1 mo, 2 mo post DSB *n*=4 and old *n*=3. Data shown represents the mean±s.e.m. Scale bar, 8 μM. *P* values were determined using the Kruskal–Wallis test to AdV samples followed by a *post hoc* Dunn’s test. *P<0.05, **P<0.01, ***P<0.001, ****P<0.0001. Mo, months.

When compared with both naturally aged (28-month-old) and young AdV control mice, DSB-induced young mice showed an elevated level of some, but not all, of the ageing phenotypes observed in the naturally aged mice at 1- and 2 month after DSB induction (Table 1, Fig. 2a). Most notably, hepatocyte nuclei were enlarged (karyomegaly; Supplementary Fig. 2a), inflammatory infiltrates and extramedullary haematopoiesis (Supplementary Fig. 2c) were increased. Of note, intranuclear inclusions (Supplementary Fig. 2b) trended towards significance for both 1 and 2 months post DSB, but most likely was not significant due to sample size limitations. Another ageing phenotype often found in liver of normally aged animals is lipofuscin, or pigment granules composed of oxidized lipid and protein residues of lysosomal digestion. However, while prominently present in normally aged mice, lipofuscin did not increase after DSB treatment (Table 1). Of note, young mice treated only with DOX but had not received an injection of SacI adenovirus did not display any changes in multiple pathology as compared with young, wild-type controls (data not shown). To quantify the extent of karyomegaly, we scored hepatocyte size by volumetric analysis of 4′,6-diamidino-2-phenylindole (DAPI)-stained liver sections. The results (Supplementary Fig. 2d) confirmed a statistically significant increase in nuclear size in the DSB-induced mice. Hepatocytes have the ability to undergo changes in ploidy during development and ageing^23^, which could also contribute to karyomegaly. Thus, we utilized a quantitative dual-colour interphase fluorescent *in situ* hybridization (FISH) approach to score the ploidy of hepatocytes as compared with spleen as a control for normal diploid tissue^24^. When analysing two autosomes, Chr 1 and 18, we did not observe a significant increase in the average ploidy in hepatocytes, either 1 or 2 months after DSB induction as compared with AdV controls (Fig. 2c, Supplementary Fig. 4). These results indicate that the observed increase in nuclear size in response to DSB treatment is not likely to be attributable to an increase in DNA content.

As shown by pathology analysis, portal and lobular lymphocytic infiltrates are significantly increased after DSB induction. To further test for the infiltration of inflammatory cells, we immunostained liver sections with IBA1 (also known as AIF1), a common marker used for delineating activated macrophages. The results indicated a significant increase from 1.2% to ∼3.3 and 2.8%, respectively, of infiltrating activated macrophages in 1- and 2-month post-DSB livers as compared with young AdV control mice (Fig. 2b; Supplementary Fig. 3). An increase in activated macrophages to ∼3% was also found in normally aged livers. These data suggest that white blood cells, either myeloid or lymphocytic, can infiltrate liver in response to DSBs, a putative cause of the age-related increase in inflammation.

Mitochondrial fusion is a well-documented ageing phenotype, originally characterized in ageing human hepatocytes^25^. Given the potentially deleterious effects of DSBs and the well-documented role of mitochondrial defects in ageing^26^,^27^, we chose to analyse mitochondrial volume after DSB treatment, using immunofluorescent staining of TOM20, a component of the mitochondrial outer membrane complex responsible for shuttling in mitochondrial pre-proteins^28^. Mitochondrial volume was found to be increased from 0.48 μm^3^ in AdV controls to 0.81 μm^3^ and 0.64 μm^3^ in 1- and 2-month post-DSB livers, respectively (Fig. 2d). In keeping with previous studies^25^, we also found a drastic increase in volume in our aged cohort to 0.98 μm^3^. Thus, our data show that DSBs alone can significantly affect mitochondrial size, yet another, well-documented ageing phenotype.

**Figure 3:**
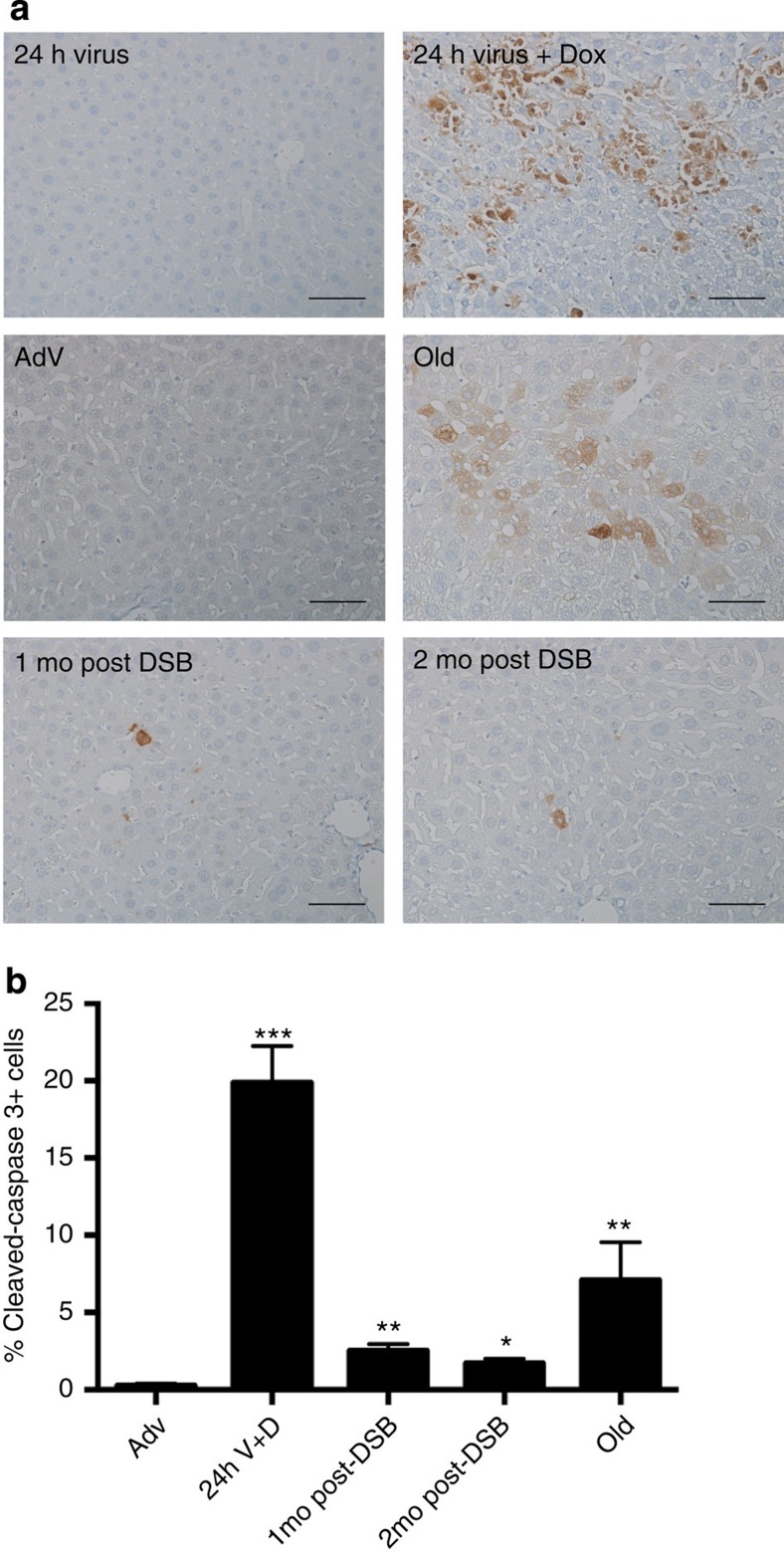
Induction of apoptosis after DSB treatment. (**a**) Liver sections were immunostained for cleaved caspase-3 after SacI adenoviral tail-vein injection as indicated. Magnification, × 20. Scale bar, 100 μM. (**b**) Quantification of cleaved caspase-3 from three images per *n*, where AdV, 1 mo, 2 mo post DSB *n*=4, 24 h V+D *n*=2 and old *n*=3. *P* values were calculated using the Kruskal–Wallis test to AdV samples followed by a *post hoc* Dunn’s test. *P<0.05, **P<0.01, ***P<0.001. Mo, months.

A major cellular hallmark of ageing in liver as well as in other organs is an increase in apoptotic cells^29^. Using immunostaining for cleaved caspase-3 as a measure of apoptosis, we observed a marked increase in apoptotic cells in 28-month-old, normally aged livers as compared with young, AdV control animals (Fig. 3), confirming findings by others^29^,^30^. When we stained for cleaved caspase-3 at 24 h after the first Sac1 AdV treatment, a large per cent of apoptotic cells, ∼20%, were present relative to 24-h AdV control mice (Fig. 3). Notably, we still observed an elevated per cent of apoptotic cells in livers from mice 1- and 2 months after DSB induction, compared with livers from young adenoviral control mice (Fig. 3). These findings indicate that as late as 2 months after DSB induction, the number of apoptotic cells remains elevated. It is conceivable that this reflects delayed apoptosis, for example, due to further accumulation of damage.

**Figure 4:**
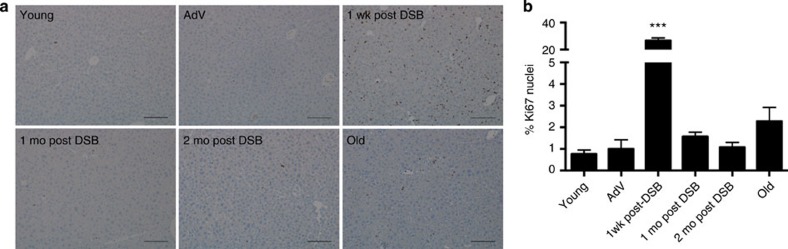
Cell proliferation after DSB treatment. (**a**) Liver sections were immunostained for Ki67 after SacI adenoviral tail-vein injection as indicated and images acquired at × 10 magnification. Scale bar, 150 μM. (**b**) Quantification of the per cent of Ki67+ nuclei of total nuclei from three images per *n*, where AdV, 1 mo, 2 mo post DSB *n*=4, 24 h V+D *n*=2 and old *n*=3. Data represent the mean±s.e.m. *P* values were determined using Student’s unpaired *t*-test. ***P<0.001. Mo, months; wk, weeks.

As suggested by the increase in apoptosis after the first wave of DSB induction, it is likely that somatic stem cell compartments are activated to stimulate liver regeneration. To confirm that cell proliferation occurred in these livers, we immunostained for a well-known nuclear proliferation marker, Ki67, shortly after DSB induction. We observed a wave of proliferation, ∼26% Ki67+ nuclei, 1 week after the first DSB induction (Fig. 4). However, at 1 and 2 months after DSB induction, signs of significant proliferation had subsided (∼1.6 and 1.1%, respectively) to levels almost similar to that of control mice, ∼1%. These data indicate that the mouse liver undergoes robust regeneration after the first wave of DSBs, most likely to repopulate the great number of cells lost by apoptosis. We also observed a slight increase in Ki67 staining in 28-month-old normally aged mice, although this increase was not statistically significant. However, it is tempting to speculate that such a late increase in cellular proliferation reflects the regeneration of spontaneously damaged liver tissue that occurs even at old age.

### DSBs induce markers of cellular senescence

**Figure 5:**
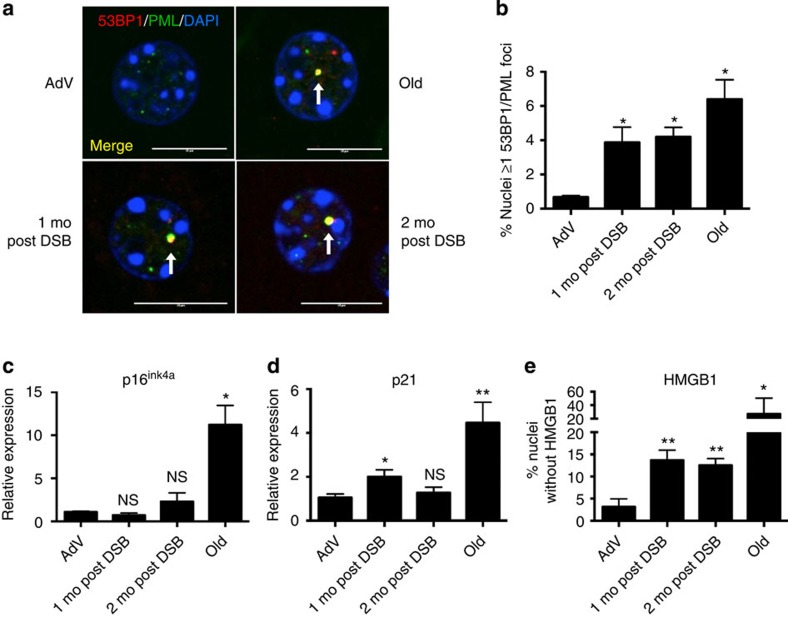
DSBs trigger senescence in the mouse liver. (**a**) Representative images of DNA-SCARS analysed by confocal microscopy; 53BP1 (red), PML nuclear bodies (green) and DAPI (blue). White arrows indicate DNA-SCARS (co-localized PML and 53BP1). Scale bar, 10 μM. (**b**) Quantification of nuclei with ≥1 co-localized 53BP1 and PML focus. Data shown are the mean±s.e.m. of all biological replicates for each cohort, where AdV, 1mo, 2mo post DSB *n*=4 and old *n*=3; >300 nuclei were scored per *n*. (**c**,**d**) Quantitative real-time PCR (qPCR) analysis of (**c**) p16^ink4a^ and (**d**) p21 expression; relative expression was calculated using the ΔΔ*C*t method and normalized to GAPDH expression levels. (**e**) Liver sections were immunostained for HMGB1 and scored as the per cent nuclei lacking HMGB1 staining. For **c**–**e**, data shown represent the mean±s.e.m. of all biological replicates for each cohort, where AdV, 1 mo, 2 mo post DSB *n*=4 and old *n*=6 for each cohort and for **e** >1,000 nuclei were scored per *n*. *P* values were calculated using Student’s unpaired *t*-test except for **b**, where the Kruskal–Wallis test to AdV samples followed by a *post hoc* Dunn’s test was performed. *P<0.05, **P<0.01, NS, not significant.

In addition to an increase in apoptotic cells, senescent cells have also been found at an increased frequency in aged tissues^8^,^10^. Indeed, cellular senescence is now considered a major hallmark of *in vivo* ageing^31^. Senescent cells are well characterized by persistent foci of γ-H2AX and 53BP1, markers of DSBs^10^,^32^. The 53BP1 foci in senescent cells are termed DNA segments with chromatin alterations that reinforce senescence (DNA-SCARS)^13^, and are characterized by the co-localization with promyelocytic leukemia (PML) nuclear bodies. Thus, we immunostained for 53BP1 and PML (Fig. 5a) 1 and 2 months after DSB induction, and compared 53BP1-PML co-localization with young AdV control and naturally aged mice. Mouse livers 1 and 2 months after DSB induction showed a significant increase in the number of nuclei with DNA-SCARS (4.2% and 3.9% of total hepatocytes, compared with 0.7% in AdV controls; Fig. 5b). The long-term presence of DNA-SCARS 2 months after DSB induction attests to the persistent nature of DSBs. In addition, we show for the first time that DNA-SCARS increase in the liver of normally aged mice (6.4% of total hepatocytes).

To confirm and further characterize DSB-induced cellular senescence, we examined three senescence markers: expression of *p16*^*ink4a*^, *p21* (ref. 33), and loss of nuclear high-mobility group box 1 (HMGB1)^34^. We found no statistically significant change in p16^ink4a^ expression at 1 or 2 months after DSB induction (Fig. 5c). However, in concordance with previous studies^33^, we did observe a significant increase in *p16*^*ink4a*^ expression in the livers of normally aged animals (Fig. 5c). By contrast, *p21* expression increased approximately twofold 1 month after DSB Induction, followed by a slight decline 1 month later (Fig. 5d). *p21* expression was also increased, ∼4.5-fold, in normally aged livers, as previously reported^35^,^36^. Recently, the loss of nuclear HMGB1, a chromatin associated protein, and its secretion was shown to be a hallmark of many senescent cells^34^. HMGB1 is an alarmin, a dual function protein that can promote inflammation^37^,^38^. Immunostaining for HMGB1 showed significant loss of nuclear HMGB1 in livers 1 and 2 months after DSB induction (∼14 and ∼13%, respectively; Fig. 5e). Normally aged livers also showed a significant loss of nuclear HMGB1 (∼20% compared with ∼3% for young controls), but as with many ageing characteristics there was considerably high variation between aged animals. With the exception of *p16*, an increase of which could not be detected, these results point towards an accumulation of senescent cells in response to DSBs.

### DSBs induce age-related changes in gene expression profiles

Because global gene expression profiles are excellent biomarkers of ageing^30^,^39^,^40^, we compared the gene expression patterns in livers from normally aged mice and in mice at 1 and 2 months after DSB induction with those in livers from young AdV control mice. To date, most gene expression studies of ageing have utilized microarrays. To more sensitively detect potential similarities between normal and DSB-induced liver ageing, we used the more powerful method of RNA-seq. We sequenced directional libraries of liver from three young mice, two young AdV controls, three normally aged mice and three mice at 1 and 2 months after DSB induction to an average depth of ∼30 million paired-end reads. Reads were then aligned using GSNAP^41^, with >80% mapping to the reference mouse genome (Supplementary Table 1). We assembled and counted transcripts using the Python-based programme HTSeq, and called differentially expressed transcripts using DESeq, which utilizes a negative binomial test for calling differential expression between samples^42^. Because expression differences during ageing tend to be subtle^43^, we refrained from setting an arbitrary cutoff for fold-changes, allowing us to detect all possible effects of DSBs. In addition, we performed two-dimensional principal component analysis after sample normalization and variance stabilization transformation to ensure no technical batch effects were present (Supplementary Fig. 5).

**Figure 6:**
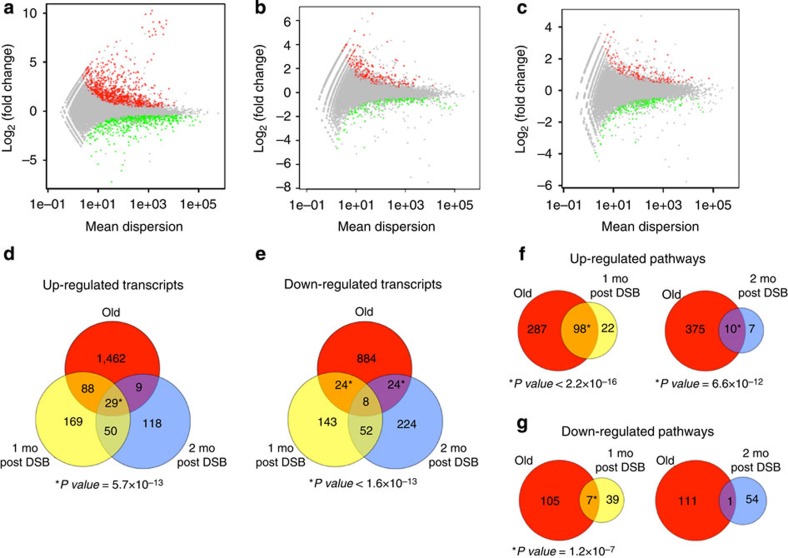
RNA-seq gene expression profiles of mouse liver after DSB induction. Differential expression plots of the normalized mean count dispersion of transcripts versus normalized log_2_(fold change) for (**a**) young versus old, (**b**) AdV versus 1 mo post DSB and (**c**) AdV versus 2 mo post DSB. *P* value cutoff is 0.05 for transcripts either significantly upregulated (red) or downregulated (green). (**d**–**g**) Venn diagrams. (**d**) Upregulated or (**e**) downregulated transcript overlaps of old, 1 mo post DSB and 2 mo post DSB as compared with their respective controls. (**f**,**g**) Significantly differentially expressed transcripts were subjected to GO analysis for biological process using DAVID. (**f**) Overlap of 1 mo post DSB or 2 mo post DSB significantly upregulated or (**g**) downregulated pathways, compared with old. *P* value of overlaps were determined using a binomial distribution test for 37,310 annotated transcripts and 13,301 GO terms for biological processes at time analysis was performed.

A total of 2,528 transcripts were differentially expressed in liver from normally aged mice, relative to young mouse liver, with 1,588 of these transcripts being upregulated and 940 downregulated (Fig. 6a). After comparing liver from young, AdV control mice to 1 month post-DSB livers, 563 transcripts were found differentially expressed, with 336 being upregulated and 227 downregulated (Fig. 6b). The same comparison with 2-month post-DSB mice showed 514 differentially expressed transcripts, with 206 being upregulated and 308 downregulated (Fig. 6c).

We analysed transcript patterns from these three comparisons to assess the overlap between normal and DSB-induced ageing. For upregulated transcripts, the 1-month post-DSB livers showed more overlap with normal old mouse liver than the 2-month post-DSB livers, with a small, but highly significant overlap of 29 genes that were upregulated in all three groups (Fig. 6d). Interestingly, comparing the overlap of downregulated genes, both 1- and 2-months post-DSB mice independently showed significant overlap with old mice, but the combined transcriptional overlap was minimal and thus did not reach significance (Fig. 6e). Together, these results indicate that DSBs significantly contribute to but do not fully recapitulate the gene expression pattern of normally aged liver.

We performed gene ontology (GO) analysis on all differentially expressed transcripts from all three groups using DAVID (Database for Annotation, Visualization and Integrated Discovery)^44^,^45^. For 1- and 2-month post-DSB livers, this resulted in significant overlap for 98 and 10, respectively, biological processes with those upregulated by normal ageing (Fig. 6f). Many of these upregulated pathways are enriched for inflammatory and cell signalling processes (Table 2), including the immune response (GO term 0006955), leukocyte proliferation (GO: 0070661) and chemotaxis (GO: 0006935), which corroborates previous findings from meta-analyses of gene expression profiles of aged liver^43^. Interestingly, regulation of apoptosis (GO: 0042981) in the 1-month post-DSB livers also significantly overlapped with normal ageing, confirming our result of delayed apoptosis after DSB induction (Fig. 3). Other significantly upregulated pathways of interest, which overlap between DSB-induced and normal ageing in liver, are cell adhesion (GO: 0007155) and regulation of actin filament polymerization (GO: 0030833), further providing evidence that DSBs induce a broad spectrum of normal age changes.

For pathways downregulated with normal ageing, we found distinctly less overlap, with seven and one GO terms for 1- and 2-month post-DSB livers, respectively (Fig. 6f). Theses significantly downregulated pathways found to overlap between DSB-induced and normal liver ageing were enriched for metabolic processes (Table 2), including monosaccharide (GO: 0005996), steroid metabolism (GO: 0008202), glucose metabolic process (GO: 0006006) and lipid biosynthetic process (GO: 0008610), also seen by others^30^. This may point towards functional impairment of normal metabolism in both DSB-induced and normal ageing.

These results are the first evidence that DSBs induce a broad spectrum of the same changes in transcript profile that also occurs during normal ageing of the mouse liver.

## Discussion

Taken together, our results indicate that DSBs alone are sufficient to cause part of the normal ageing process in mouse liver. This finding is in keeping with the indirect evidence for DSBs as a potential driver of ageing^7^,^9^,^46^. Specifically, our data show that DSBs (1) induce most but not all pathological lesions in the livers of young animals that are normally only seen at old age; (2) result in increased apoptosis, cellular senescence and mitochondrial fusion *in vivo*, generally considered as part of the normal ageing process; and (3) induce broad alterations in transcriptional expression profiles that overlap with many of the changes observed during normal ageing of the liver, which confirm previously observed results obtained by others using microarrays. Of note, our observation of DNA-SCARS, never before reported in normally aged tissue, together with the evidence that DSBs induce cellular senescence^32^,^47^, strongly suggests a primary role for DSBs in the normal ageing process. We show that DSBs *in vivo* directly affect mitochondrial structure, which is in keeping with previous observations of mitochondrial defects in DNA repair-deficient mice^48^,^49^.

We conclude that DSBs can drive some aspects of the normal ageing process, a conclusion that until now has been elusive due to the lack of models that allow studying ‘clean’ DNA lesions, independent of possible side effects associated with genotoxic agents or progeroid human and mouse syndromes. The striking similarity between DSB-induced ageing and its normal counterpart, at least in the liver, is remarkable given the great age difference between the treated young mice and the aged control group. Indeed, one might expect that many other aspects of normal ageing, dependent on the extended time period of normal mouse life span and not necessarily due to DSBs, would confound the phenotypes found associated with DSB-induced ageing so early in life.

Here we analysed only the liver, as the liver is known to be by far the main target after tail-vein injection (Fig. 1c, Supplementary Fig. 1)^50^. We also note that the side effects of SacI AdV treatment are minimal. As shown by the AdV controls, the DSB-inducing construct itself is not immunogenic and, in contrast to radiation or gene knockouts, side effects beyond the DSBs themselves are wholly absent.

Perhaps the strongest evidence that DSBs are capable of inducing a broad spectrum of normal age-related changes already at very early age is the significant overlap between gene expression profiles. This overlap in altered transcript profiles is not complete, which could be due, at least in part, to stochastic variation between animals, which in aged mice can be considerable^51^. This may be responsible for some real overlap not reaching statistical significance. Indeed, the 1- and 2- month post-DSB patterns also differ from one another. However, it is conceivable that other, non-DSB, pro-ageing factors play a role. For example, DSBs alone are apparently insufficient to accelerate lipofuscin accumulation. In this respect, it is likely that the accumulation of protein aggregates and other age-related alterations in biological macromolecules drive aspects of the ageing process independent of DNA damage^52^. We also noted the differences between normal and DSB-induced ageing in cellular senescence markers. For example, *p16*^*ink4a*^ expression was found by us and others^33^ to increase significantly in normally aged liver, while no changes were observed in DSB-induced livers from young animals. It is plausible that the fraction of senescent cells in the DSB-induced livers was too low to detect a significant increase in *p16*^*ink4a*^. On the other hand, increased *p21* as well as loss of nuclear HMGB1 were observed in both normal and DSB-induced, premature ageing.

By what mechanism do DSBs induce distinct ageing pathologies? One mechanism could be apoptosis. As we showed, an elevated rate of apoptosis persists at least 2 months after DSB induction, possibly affecting liver regenerative capacity. While cell loss alone is unlikely to explain all the aspects of ageing, widespread atrophy is certainly associated with normal ageing. Second, DSBs can cause cellular senescence, which is emerging as a possible major cause of ageing *in vivo*^32^,^53^. Senescence, despite being a safeguard against cancer, might cause age-related degeneration not only through a decrease in mitotic potential, thereby reducing regenerative capability, but also through the secretion of inflammatory cytokines^54^, which can even promote hyperplastic growth in surrounding cells^47^. Our observations of increased DNA-SCARS and loss of HMGB1 indicate an accumulation of senescent cells in response to DSBs *in vivo*. Furthermore, we provide evidence that clean DSBs can induce senescence, without persistent *p16*^*ink4a*^ expression, and potentially dependent on a p53 response based on the observed increased in *p21* and nuclear export of HMGB1. These markers, especially *p21*, may sustain the senescent state until further, irreversible phenotypes develop, such as changes in the epigenetic landscape^55^. This may explain why we see a decrease in *p21* 2 months after DSBs. While nuclear loss of HMGB1 may not fully cause a senescent state, its mechanism as a pro-inflammatory alarmin once exported out of the nucleus and ultimately out of the cell may partially cause the increase in activated macrophage infiltration due to its known interaction with IL-1β (ref. 38), thus reinforcing the SASP/inflammatory senescent phenotype regardless of *p21* and *p16*^*ink4a*^ expression. These results coupled with the increase in infiltrating lymphocytes and macrophages may explain the observed increase in inflammatory gene expression patterns. Moreover, previous studies have shown that unique respiratory bursts in macrophages can cause DNA damage in surrounding cells^56^. Thus, our evidence for persistent DNA damage and DNA-SCARS up to 2 months after the DSB treatment may well be sustained or even caused by the observed increase in infiltrating activated macrophages. Alternatively, the reduction in *p21* 2 months after DSB induction may reflect the clearance of pre-malignant senescent hepatocytes by the immune system^57^.

A third possible mechanism that could underlie the observed broad spectrum of gene expression profile changes is the demonstrated effect of DNA damage on metabolism. Indeed, similar alterations in lipid metabolism as we observed after DSB-induced ageing have been reported for mouse models with defects in DNA repair^22^,^58^.

Finally, when erroneously repaired, DSBs alter the genome or epigenome, which could explain some of the gene expression changes observed during both normal and DSB-induced ageing^59^.

In summary, our data upon the molecular and cellular phenotypes triggered in mice on the induction of clean DSBs, without side effects due to protein or lipid damage, enables—for the first time—the dissection of the component of ageing that could be due to a defined DNA lesion alone. In keeping with indirect data on the likely pro-ageing effect of DSBs, we show that DSBs can explain some of the chronic symptoms associated with physiological ageing. Interestingly, our observation that ageing symptoms are less severe at 2 months as compared with 1 month after treatment suggests that some of these phenotypes are reversible. To some extent, this may be due to the short-term nature of the DSB induction. At such an early age, cell and tissue regenerative capacity is still high. However, similar phenotypes at old age may not be as reversible. Indeed, ageing is associated with a progressive decline in stem cell function, resulting in less-effective tissue regeneration^60^. While as yet we do not know the long-term effects of our DSB treatment, these could be similar to what is observed in adult survivors of pediatric cancer. Patients treated with harsh clastogens early in life see most adverse phenotypes disappear several months after the cessation of treatment only to return decades later, eerily mimicking premature ageing^16^,^17^. The experimental system described here should allow the systematic assessment of the long-term effects of DSBs in mice across tissues and test interventions to promote cell and tissue rejuvenation at different ages.

## Methods

### Adenoviral stock production

HEK 293A/tTS cells were cultured in DMEM supplemented with 10% fetal bovine serum, 1% MEM non-essential amino acids, 4 mM L-glutamine, 1% penicillin–streptomycin, 1% sodium pyruvate. Adenoviral stocks, pAd/Tight-SEV5-CMV-rtTA (A/TSCR), were mass produced in HEK 293A/tTS cells by infecting cells with crude A/TSCR virus at a multiplicity of infection of 1. Cells were then incubated until a cytopathic effect of 90% was reached. Adenovirus was purified according to the Adeno-X Mega Purification Kit protocol (Clontech) and resuspended in 1 × PBS.

### Animals and tissue collection

All procedures involving animals were approved by the Institutional Animal Care and Use Committee (IACUC) of Albert Einstein College of Medicine. Three male Balb/C mice of age 27 months were obtained from the NIA (naturally aged mice), and allowed to recover 1 month after delivery before killing. Male, 8-week-old experimental and control mice were also obtained from the same NIA colony. When mice reached 12 weeks of age, DOX (2 mg ml^−1^ in 5% sucrose solution) was added to the drinking water 24 h before receiving a tail-vein injection of 0.2 ml of A/TSCR virus. Mice were removed from DOX 24 h after the last tail-vein injection. Mice were killed at the indicated intervals (24 h, 1 week, 1 month, 2 months post DSB) thereafter, liver was harvested and immediately fixed in 10% phosphate-buffered formalin for up to 48 h or flash frozen and kept at −80 °C. Formalin-fixed tissues were paraffin embedded and 4.5 μm sections were cut and used for immunohistochemistry and immunofluorescence.

### Immunohistochemistry and immunofluorescence

Formalin-fixed paraffin embedded sections were deparaffinized, rehydrated, incubated in 0.01 M citrate buffer (pH 6.0) at 95 °C for 20 min and washed in 1 × PBS according to standard protocols. Primary antibodies were anti-Histone H2A.X-S139ph at 1:1000 (Active Motif), anti-cleaved caspase-3 (Asp175) at 1:50 (Cell Signaling), anti-Ki67 at 1:400 (Dako), anti-IBA1 at 1:1,500 (Wako). Secondary antibody was HRP-conjugated goat anti-rabbit (Santa Cruz Biotechnology) at 1:1,000. HRP activity was detected using a DAB substrate kit (Invitrogen). Liver sections were stained for pathological analysis according to established protocols using haematoxylin and eosin. For lipofuscin analysis, liver sections were deparaffinized, rehydrated and mounted with mounting medium. Slides were then exposed to ultraviolet light and autofluorescent molecules (lipofuscin) assessed by a pathologist and given a score between 0 and 5 to correspond to ageing pathologies. For immunofluorescence, liver sections were stained with anti-53BP1 (Bethyl Laboratories) at 1:500, anti-PML 36.1–104 (Millipore) at 1:50, anti-HMGB1 at 1:2,000 (Abcam) and anti-TOM20 clone 2F8.1 (Millipore) at 1:500. Secondary antibodies were AlexaFluor 488 donkey anti-mouse and AlexaFluor 594 donkey anti-rabbit (Invitrogen) at 1:1,000. Slides were counterstained with sudan black, mounted with ProLong Gold Antifade reagent with DAPI (Invitrogen) and allowed to cure overnight at room temperature.

### Microscopy and analysis

Immunofluorescence images were acquired using a Leica SP5 laser scanning confocal microscope and a 40 × oil objective with pinhole set to 1 AU and line average set to 3, and analysed using Volocity software; background thresholds were set based on young adenoviral control samples and antibody control samples. Immunohistochemistry images were acquired using a Nikon CoolScope at 10 or 20 × magnification and analysed using ImageJ software. HMGB1 immunofluorescence images were quantified using CellProfiler, an open-access image analysis programme ( www.cellprofiler.org).

### Fluorescent *in situ* hybridization

Tissue FISH was performed according to a prior protocol and adapted for two-colour chromosome labelling^24^,^61^. The BAC clones used were as follows: RP23–34K7 (Chr.1qA1) and RP23–16K15 (Chr.18qE1). Probes were labelled by nick translation using spectrum orange-dUTP and DY-415-dUTP. Liver sections were treated using Vysis Paraffin Pretreatment Reagent Kit (Abbott Molecular Inc., 02J02–032) with some modifications. In brief, FFPE sections were baked at 56 °C overnight on a slide warmer, deparaffinized by Citisolve (Thermo-Fisher) for 3 × 10 min, dehydrated in 100% EtOH for 3 × 10 min at room temperature, and then air-dried. The slides were then treated with pretreatment buffer for 30 min at 80 °C, washed with wash buffer for 5 min at room temperature, and then in purified water for 1 min. Slides were immersed into protease solution for 18 min at 37 °C, washed with purified water for 1 min, then wash buffer for 5 min at room temperature. We dehydrated slides in a series of 70, 90, 100% ethanol. Co-denatured slides and probes at 76 °C for 5 min; hybridized slides at 37 °C overnight. Slides were placed into 2 × saline-sodium citrate with 0.4% NP40 solution for 2 min at 72 °C for detection and then air-dried. Slides were mounted with ProLong Gold Antifade reagent with DAPI (Invitrogen). FISH images were acquired using a Zeiss Axiovert 200 at × 400 magnification.

### qPCR

Total RNA was converted into cDNA using SuperScript III First-strand Synthesis Kit (Invitrogen) and 50 ng of random hexamers. Quantitative PCR was performed using 100 ng of cDNA and ABI StepOne Plus system for TaqMan (ABI) assays. All calculations were performed using the ΔΔ*C*t method, with TaqMan assays normalized to GAPDH and biological replicate values representing the mean of technical triplicates. Primer and probes are as follows: p16^Ink4a^ forward 5′-CCCAACGCCCCGAACT-3′, reverse 5′-GCAGAAGAGCTGCTATGTGAA-3′, probe 5′-TTCGGTCGTACCCCGATTCAGGTG-3′; p21 assay ID Mm04205640_g1; mouse GAPDH assay ID Mm99999915_g1.

### Directional RNA sequencing

Flash-frozen tissues were homogenized with 1.4 nm ceramic bead matrix and Trizol (Invitrogen) using the MP FastPrep-24 system. Total RNA quality was checked on an Agilent 2100 Bioanalyzer; only samples with a RNA Integrity Number greater than 8.5 were used for subsequent analysis. Total RNA was treated with DNaseI, column purified using the miRNeasy Mini Kit (Qiagen), and depleted of ribosomal RNA with Ribo-Zero Magnetic Gold Kit (Epicentre), followed by ethanol precipitation. Depleted RNA was converted to cDNA using SuperScript III First-Strand Synthesis Kit (Invitrogen) with 80 ng random hexamers and 50 μM oligo dT and subsequently ethanol precipitated. Single-stranded cDNA was converted to dsDNA by DNA polymerase I while incorporating dU/VTPs (10 mM). Samples were fragmented in 1 × TE to 200–300 bp using Covaris. After fragmentation, samples were purified using the MinElute PCR purification kit (Qiagen). Fragmented samples underwent standard end-repair, dA-tailing and adapter ligation using Illumina TruSeq adapters for multiplexing. Adapter-ligated cDNA was treated with uracil-DNA glycosylase followed by enrichment PCR using Q5 polymerase (New England Biolabs) for 18 cycles. Libraries were size selected for 150–600 bp on a 2% low-melt ultra low-range agarose gel stained with SYBR Gold (Invitrogen) to eliminate adaptor dimers. Purified libraries were clustered (seven samples per flow cell lane) and sequenced on an Illumina HiSeq2000 for 100 bp paired-end reads.

### Gene expression analysis

Pass filter sequences were aligned with GSNAP v2013-01-23 according to default settings with novel splicing, using the mm9_all reference genome^41^. Counts were generated for each sample using HTSeq v0.5.3p9. Combined HTSeq counts were analysed using DESeq v1.10.1 (ref. 42) in RStudio v0.97.312. Significant differentially expressed genes were entered into DAVID v6.7 for pathway and GO enrichment analysis using an EASE score of 0.05 with a gene count minimum of 3 for each GOTERM_BP_FAT^45^. A Fisher’s exact test value below 0.05 was considered statistically significant for GO pathway analysis. *P* value of overlap was determined using binomial distribution test.

### Statistical methods

Statistical analyses for experiments not involving RNA-seq data were performed either in R v2.15.2 using RStudio v0.97.312 or in GraphPad Prism 6. For most analyses (unless otherwise stated), the mean value is shown with the s.e.m. of biological replicates. *P* values were calculated using tests for parametric, Student’s unpaired *t*-test, or non-parametric, Mann–Whitney *U*-test. Multiple group comparisons were performed using the Kruskal–Wallis test followed by a *post hoc* Dunn’s test to correct for multiple testing. A significance value cutoff of *P*<0.05 was set for all tests.

## Additional information

**How to cite this article:** White, R. R. *et al.* Controlled induction of DNA double-strand breaks in the mouse liver induces features of tissue ageing. *Nat. Commun.* 6:6790 doi: 10.1038/ncomms7790 (2015).

## Supplementary Material

Supplementary Information Supplementary Figures 1-5 and Supplementary Table 1

## Figures and Tables

**Table 1:** Histopathological analysis of DSB-induced mouse liver.

**Pathology**	**Young**	**AdV**	**1 mo post DSB**	**2 mo post DSB**	**Old**
Karyomegaly	1.62±0.38	2.27±0.14	3.00±0*	2.25±0.25	4.00±0**
Intranuclear inclusions	0±0	0.09±0.10	0.38±0.13^#^	0.38±0.13^#^	3.00±0**
Lobular infiltrates	0.38±0.24	0.81±0.26	1.63±0.13*	1.38±0.24	3.67±0.33**
Portal infiltrates	1.12±0.12	0.90±0.09	1.25±0.15*	1.25±0.25	2.33±0.66*
EMH	0±0	0±0	0.75±0.25*	0.25±0.25	2.33±0.66*
Lipofuscin	0.37±0.12	0.20±0.13	0.25±0.14	0±0	2.0±0.29*

DSB, double-strand break; EMH, extramedullary haematopoiesis; mo, months.

Mice were killed and the livers were harvested at 1 mo or 2 mo post DSB induction. Livers were also harvested from untreated young mice (young), young SacI AdV injected mice without DOX (AdV) 1 and 2 months after injection and 28-month-old untreated aged controls (old). Liver sections were stained with haemotoxylin and eosin and assessed blinded for pathological characteristics of aging. Pathology was scored on a scale of 0–5, with 0 having absent pathology and 5 having severe pathology. Young *n*=5, AdV *n*=6, 1 mo post DSB *n*=4, 2 mo post DSB *n*=4, old *n*=3. Data shown are the mean values±s.e.m. *P* values were calculated using the Kruskal–Wallis test to AdV samples followed by a *post hoc* Dunn’s test. *P<0.05, **P<0.01. ^#^*P* value=0.06.

**Table 2:** Top overlapping gene ontology pathways.

**GO term (biological process)**	**Old**	**1 mo post DSB**	**2 mo post DSB**
*Up regulated*			
T-cell activation (GO: 0042110)	9.4 × 10^−13^	5.2 × 10^−11^	2.0 × 10^−3^
Immune response (GO: 0006955)	2.2 × 10^−18^	2.8 × 10^−8^	NA
Cell activation (GO: 0001775)	5.5 × 10^−18^	4.6 × 10^−8^	NA
Chemotaxis (GO: 0006935)	3.0 × 10^−6^	2.8 × 10^−7^	1.5 × 10^−3^
T-cell proliferation (GO: 0042098)	2.0 × 10^−6^	1.8 × 10^−6^	1.3 × 10^−3^
Integrin-mediated signalling pathway (GO: 0007229)	3.4 × 10^−4^	9.0 × 10^−6^	2.8 × 10^−3^
Lymphocyte proliferation (GO: 0046651)	3.0 × 10^−5^	2.1 × 10^−5^	4.1 × 10^−3^
Leukocyte proliferation (GO: 0070661)	3.9 × 10^−5^	2.4 × 10^−5^	4.4 × 10^−3^
Mononuclear cell proliferation (GO: 0032943)	3.9 × 10^−5^	2.4 × 10^−5^	4.4 × 10^−3^
Cell cycle (GO: 0007049)	1.0 × 10^−4^	3.2 × 10^−5^	2.0 × 10^−2^
Cell proliferation (GO: 008283)	5.1 × 10^−5^	1.5 × 10^−4^	1.2 × 10^−2^
Regulation of apoptosis (GO: 0042981)	2.0 × 10^−3^	6.3 × 10^−3^	NA
Cell adhesion (GO: 0007155)	1.8 × 10^−5^	1.8 × 10^−3^	NA
Regulation of actin filament polymerization (GO: 0030833)	1.5 × 10^−2^	3.3 × 10^−3^	NA
			
*Down regulated*			
Acute inflammatory response (GO: 0002526)	1.2 × 10^−2^	1.7 × 10^−5^	NA
Steroid metabolic process (GO: 0008202)	3.7 × 10^−3^	2.2 × 10^−4^	NA
Glucose metabolic process (GO: 0006006)	1.9 × 10^−2^	2.9 × 10^−3^	NA
Monosaccharide metabolic process (GO: 0005996)	1.6 × 10^−2^	3.2 × 10^−3^	1.0 × 10^−2^
Lipid biosynthetic process (GO: 0008610)	1.7 × 10^−6^	8.3 × 10^−3^	NA

DSB, double-strand break; GO, gene ontology; mo, months; NA, not available.

The top 10 upregulated and top five downregulated overlapping gene ontology annotations for biological processes for 1 mo post DSB or 2 mo post DSB were compared with old for upregulated and downregulated transcripts. *P* values were determined using Fisher’s exact test.
