# Supplementary material for: Controlled induction of DNA double-strand breaks in the mouse liver induces features of tissue ageing
Source: Nat Commun. 2015 Apr 10;6:6790. doi: 10.1038/ncomms7790 (PMC4394211; doi:10.1038/ncomms7790)
Supplement: Supplementary Information — Supplementary Figures 1-5 and Supplementary Table 1 [file ncomms7790-s1.pdf]

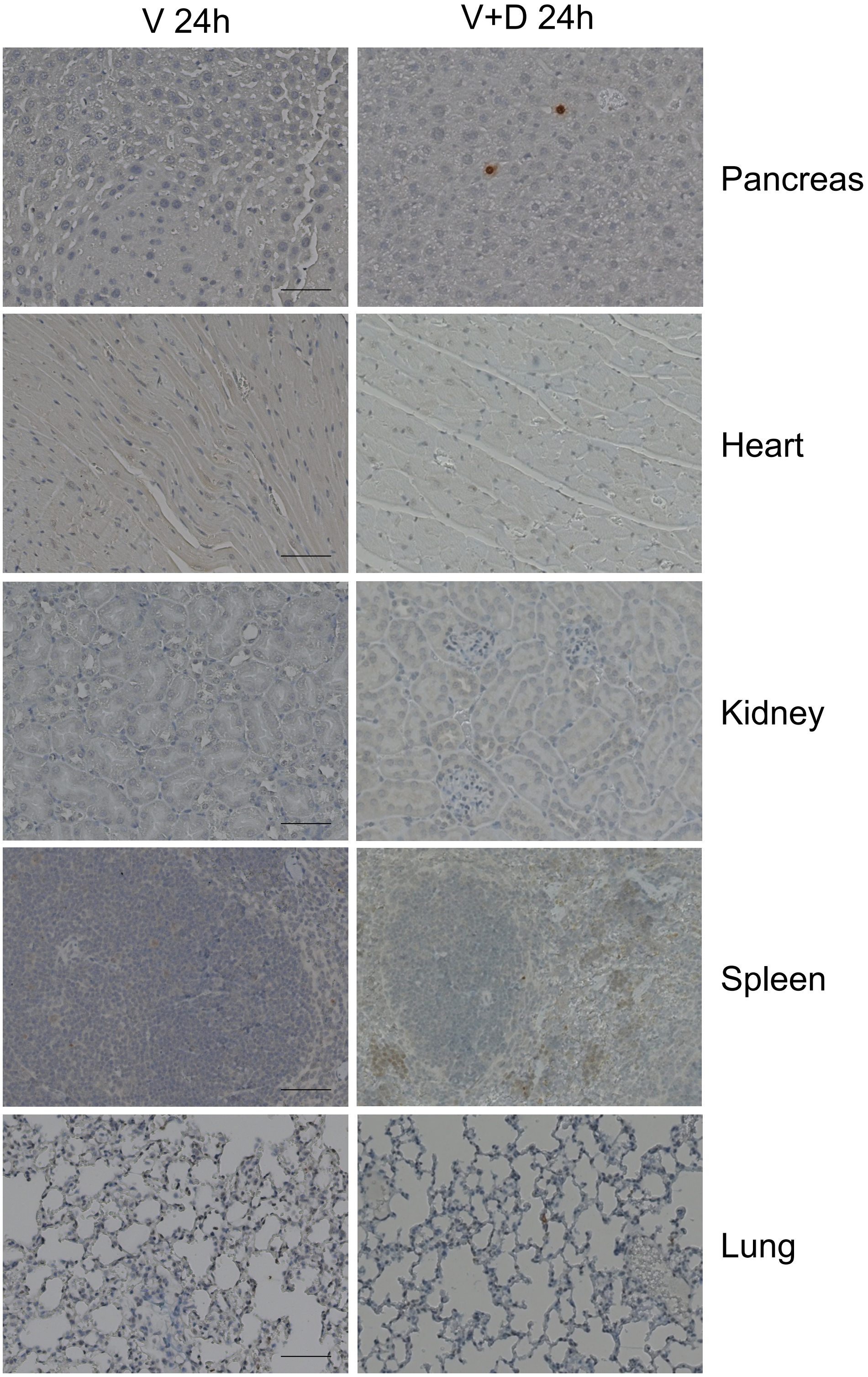

Supplementary Figure 1. γ-H2AX staining in various tissues. Tissues were harvested 24 hours after SacI adenoviral injection (V) with either no DOX or DOX (+D) in drinking water and stained for γ-H2AX. Images were acquired at 20X magnification. Scale bar = 100μM.

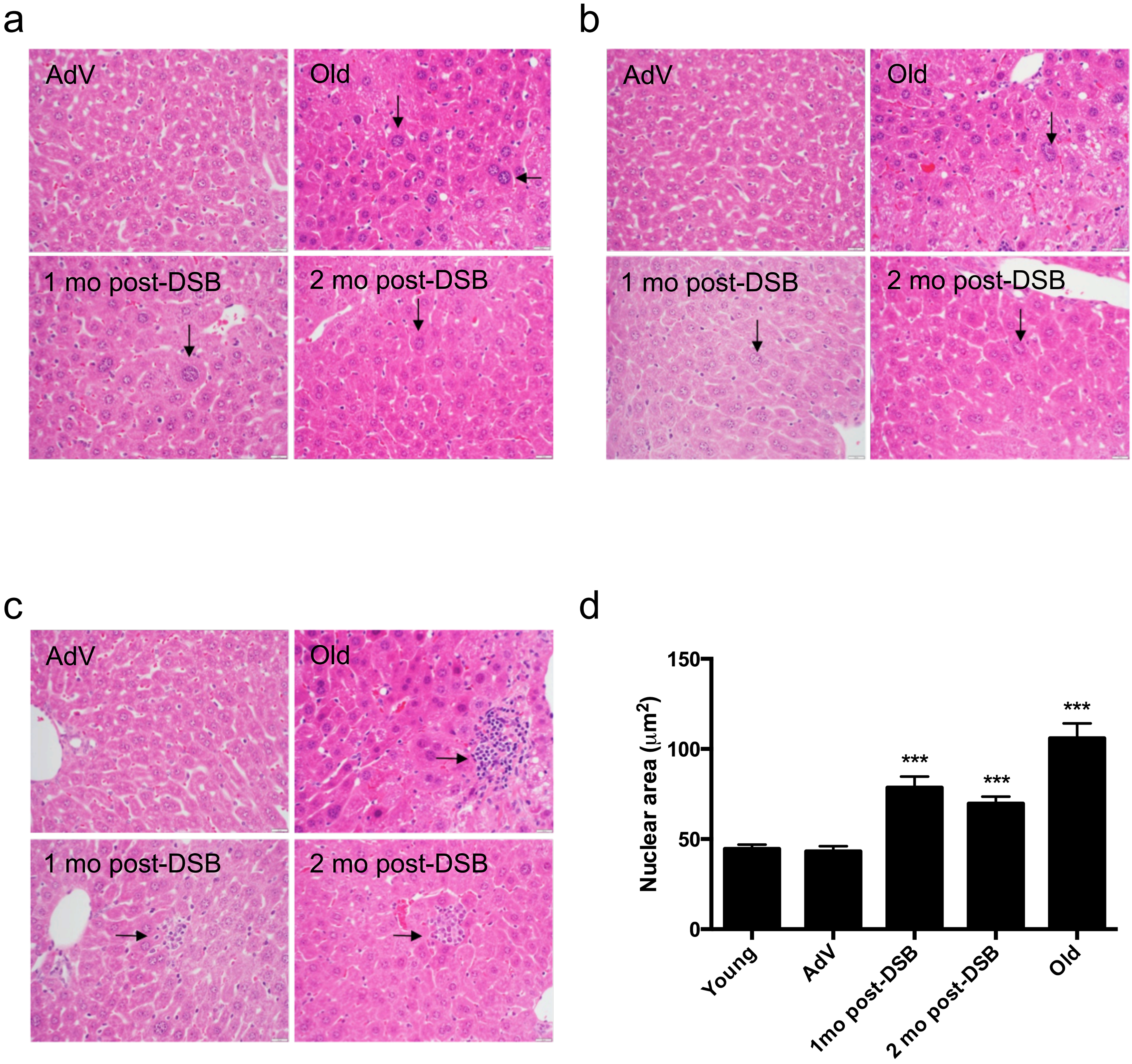

Supplementary Figure 2. DSB-induced pathologies. Liver sections were stained with H&E and normal aging pathological characteristics assessed for Table 2. Representative images for (**a**) karyomegaly (black arrows), (**b**) intranuclear inclusions (black arrows), and (**c**) extramedullary hematopoiesis (black arrows). Scale bar (white) = 20μM (**d**) Liver sections were stained with DAPI and analyzed by confocal microscopy. Nuclear area was then determined using Volocity software for 50 hepatocytes for n=3 for each cohort. Data represents mean ± s.e.m. *P* values were calculated using Mann-Whitney test to AdV samples. *** *P* <0.0001

a

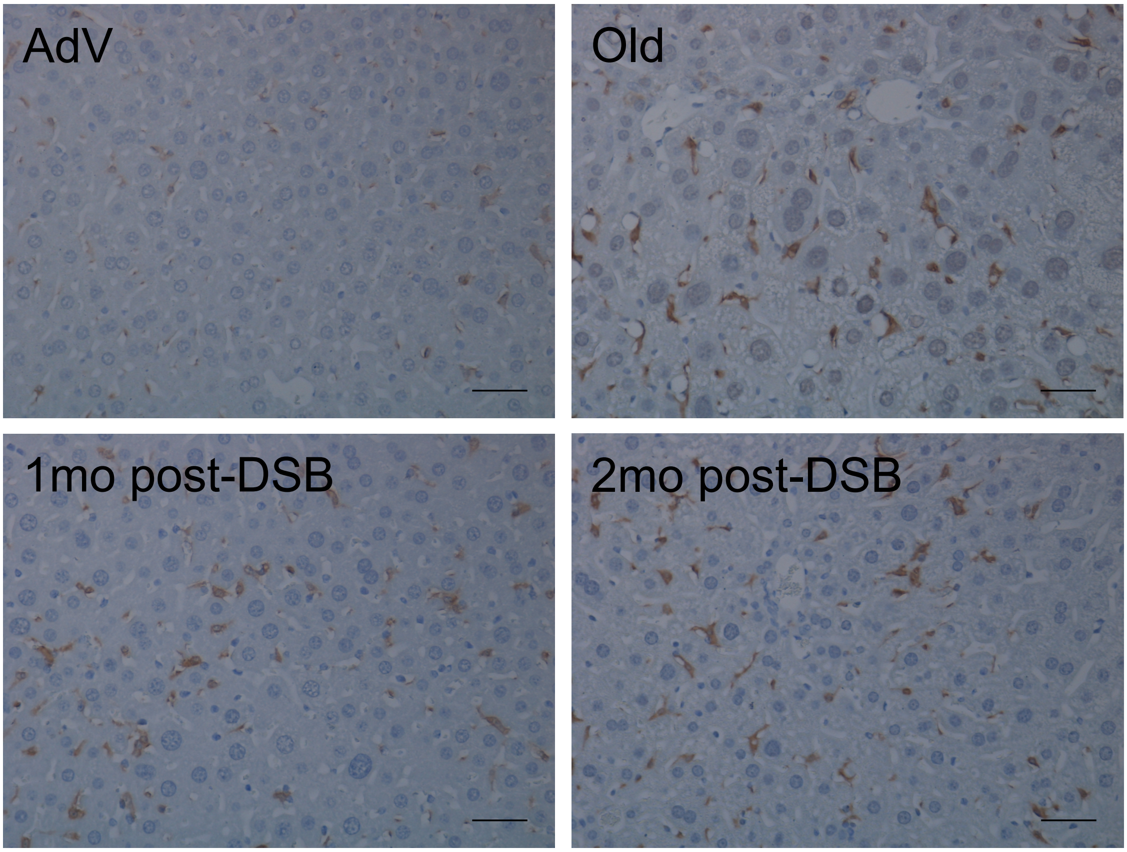

Supplementary Figure 3. Representative IBA1 staining for activated macrophages. Images were acquired at 20X magnification and analyzed for Figure 2b**.** Scale bar = 100µM.

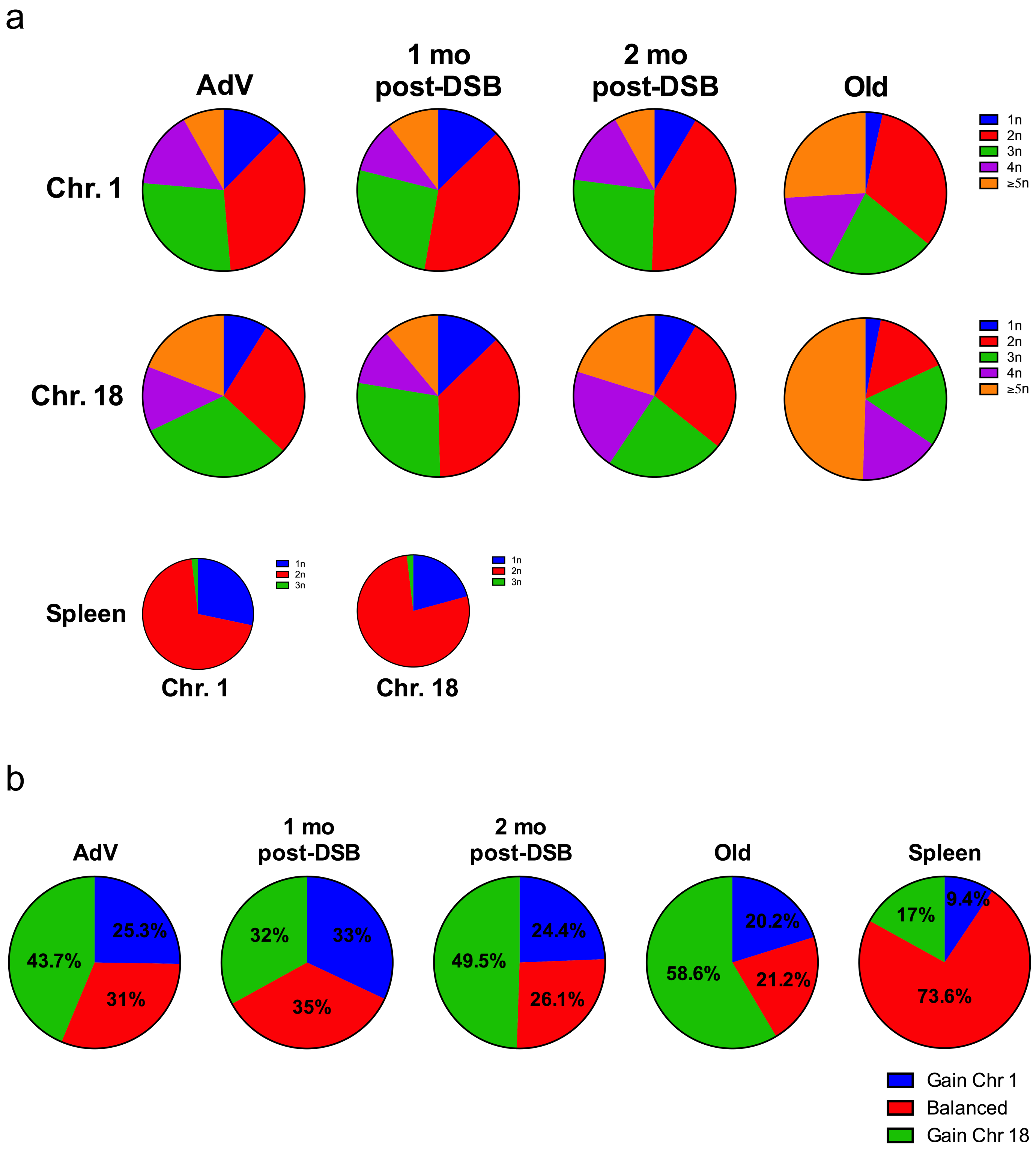

Supplementary Figure 4. Dual-Color DNA-FISH. (**a**) The spectrum of observed ploidy for Chr. 1 and Chr.18 in liver sections as assessed by the number of probes present in each hepatocyte (i.e., two Chr.1 probe signals represent 2n). (**b**) Gains in either Chr. 1 or 18, or the balance of both chromosomes was assessed and calculated as a percent of whole for each cohort (i.e., Chr1/18, 2/2 = balanced, 3/2 = Gain Chr. 1, 2/3 = Gain Chr. 18). For each cohort n=3 where >100 hepatocytes were analyzed per n, except spleen where n=1.

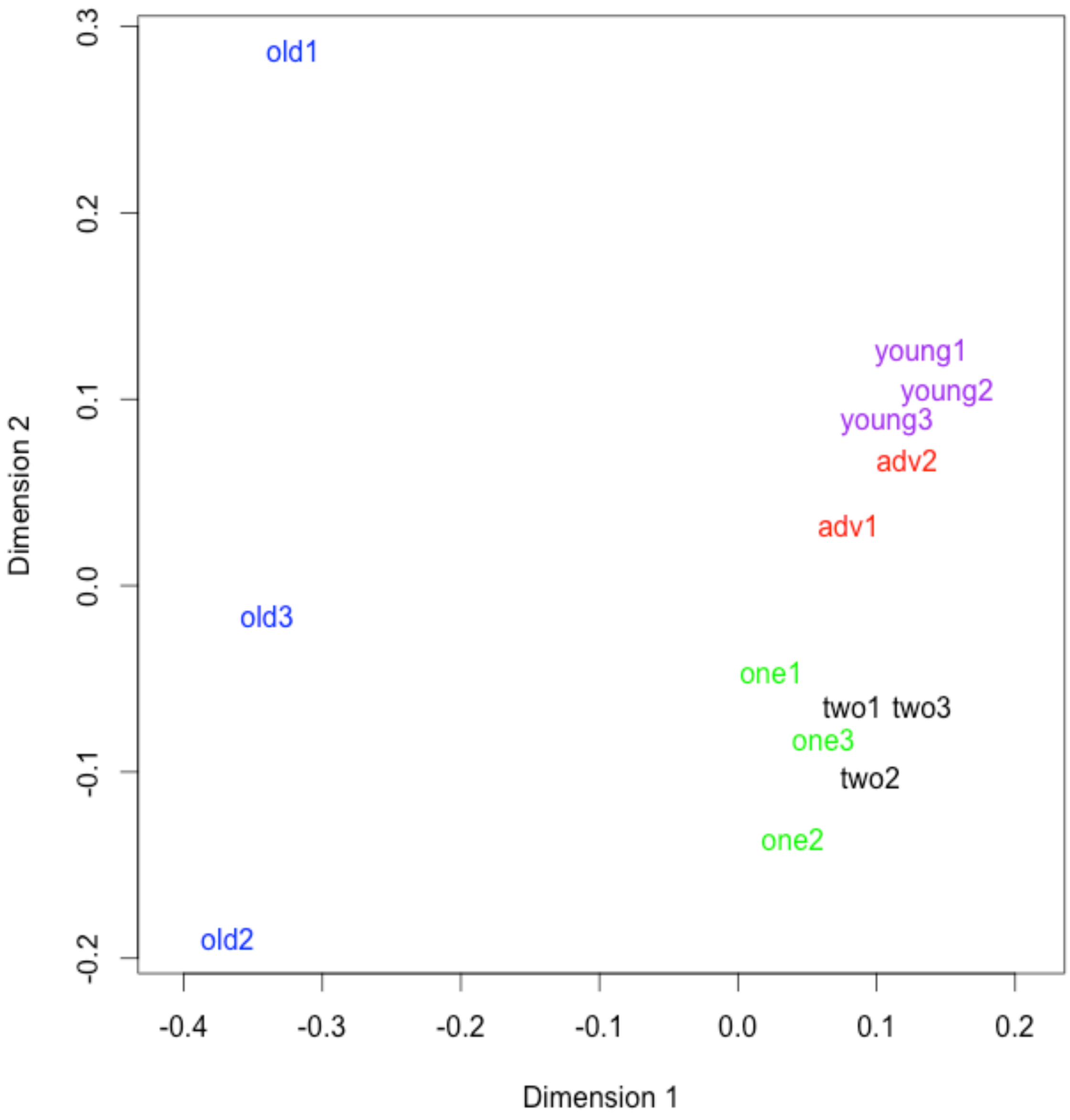

Supplementary Figure 5. Principal component analysis of biological replicates for RNA-seq. Reads from each RNA-seq sample were normalized according to library size and then variance stabilized. A principal component plot was then generated using the variance of each sample from the top 25,000 transcripts.

Supplemental Table 1. Read statistics for RNA-seq analysis.

Sample Name	Total Paired-End Reads	Percent of total reads aligned
Old 1	36,231,783	81.74
Old 2	24,838,760	89.51
Old 3	31,696,717	90.51
Young 1	24,688,962	86.71
Young 2	27,872,770	87.80
Young 3	35,410,902	85.76
AdV 1	50,799,631	85.85
AdV 2	39,795,094	89.50
1mo post-DSB 1	35,601,195	80.64
1mo post-DSB 2	28,581,527	87.31
1mo post-DSB 3	50,534,728	90.53
2mo post-DSB 1	38,980,404	90.05
2mo post-DSB 2	29,836,415	90.14
2mo post-DSB 3	32,458,851	90.51
